# Yeast and Diabetes Mellitus

**Published:** 1903-07-18

**Authors:** 


### YEAST AND DIABETES MELLITUS.

Yeast as a therapeutic remedy has figured very little in English scientific literature, but a good many observations upon its use have been made abroad, and especially in France. It is a remedy which, in the light of modern knowledge and in view of the present trend of therapeutics, certainly seems to have possibilities about it, and to be probably worthy of greater attention and study than it has hitherto received.

The latest writer on the subject, M. Boigey,[1] considers that it has a distinct and marked value as an intestinal antiseptic, and that as such its action is allied to that of naphthol, lactic acid, carbon, chalk, and bismuth. It appears to him that in the presence of the saccharomyces cerevisiæ, the elaboration of intestinal ferments is considerably decreased and the growth of microbial organisms diminished.

In discussing its mode of action he ascribes to it the power of producing increased leucocytosis, and considers that its contained organisms are not only phagocytic, but that they also produce a secretion of the nature of a bactericidal toxin. As far as the observation of the power of yeast to produce intestinal antisepsis goes, M. Boigey is confirmed in it by Debouzy and Brocq, who claim to have obtained excellent results by the use of yeast in cases of infantile diarrhœa and muco-membranous colitis of adults. These latter authors also believe that its use exercises a very marked effect of a beneficial character on the course of boils and carbuncles, while Thiercelin and Chevrey report useful results from its use as an injection in dysentery.

Boigey, however, considers that the strongest indication for the use of yeast is in diabetes mellitus, in which he was led to try it by a communication to the Bordeaux Congress in 1895 by M. Cassaet. The theoretic indications for its use in this condition depend upon the power of yeast to convert the starchy elements of food into alcohol, the invertin it contains converting the cane sugar into glucose and the glucose being changed into alcohol by the diastase. In his hands the practical results of the use of yeast have fully confirmed these theoretic views, and he considers it to be one of the most valuable remedies which are available for the treatment of this condition of pathological sugar formation.

Yeast, of course, depending as it does for its action upon the vitality of the living organisms which it contains, and at the same time being one which is administered by the mouth, stands practically by itself. Like all other remedies introduced within the intestinal tube its power of effective action is limited by the conditions that it encounters therein and the extent to which it is able to overcome them and conserve its own vitality. The extent of this vitality before introduction is therefore of great importance and inequality in the results and the unfavourable reports given by some experimenters are ascribed to insufficient care on this point. Good yeast, according to M. Boigey, should consist of 70 per cent. saccharomyces and not more than 30 of debris. He and the other authors quoted have depended on ordinary beer yeast, which normally grows at a somewhat low temperature and on a neutral medium. When introduced into the stomach therefore the duration of its survival is limited, since it undergoes digestion with a greater or less rapidity to which the amount of its secretory products and its general action inversely correspond.

Arguing from this point, Tacquemin has advocated the substitution for beer yeast of that grown from the grapes of warm countries, active at a temperature of 35 to 39 Cent. and acclimatised to an acidity equal to that of the gastric juice. In view, however, of the good results obtained by Boigey, Thiercelin and Chevrey, Brocq and Debouzy with ordinary beer yeast, it is doubtful if this more specialised product is really necessary. It is sufficient probably that the precautions prescribed by Boigey should be observed; these being that the yeast should be protected from the action of moisture and warmth, and that it should be stored in a cool dry atmosphere.

[1] Archives Gen. de Med., April 1903.

